# SARS-CoV-2 and HIV-1: Should HIV-1-Infected Individuals in Sub-Saharan Africa Be Considered a Priority Group for the COVID-19 Vaccines?

**DOI:** 10.3389/fimmu.2021.797117

**Published:** 2021-11-08

**Authors:** Wilson Lewis Mandala, Michael K. P. Liu

**Affiliations:** ^1^ Academy of Medical Sciences, Malawi University of Science and Technology (MUST), Thyolo, Malawi; ^2^ Centre for Immunology and Vaccinology, Department of Infectious Disease, Imperial College London, London, United Kingdom

**Keywords:** COVID-19, HIV, immunity, vaccine, Sub-Sahara Africa

## Abstract

Since its emergence in 2019 SARS-CoV-2 has proven to have a higher level of morbidity and mortality compared to the other prevailing coronaviruses. Although initially most African countries were spared from the devastating effect of SARS-CoV-2, at present almost every country has been affected. Although no association has been established between being HIV-1-infected and being more vulnerable to contracting COVID-19, HIV-1-infected individuals have a greater risk of developing severe COVID-19 and of COVID-19 related mortality. The rapid development of the various types of COVID-19 vaccines has gone a long way in mitigating the devastating effects of the virus and has controlled its spread. However, global vaccine deployment has been uneven particularly in Africa. The emergence of SARS-CoV-2 variants, such as Beta and Delta, which seem to show some subtle resistance to the existing vaccines, suggests COVID-19 will still be a high-risk infection for years. In this review we report on the current impact of COVID-19 on HIV-1-infected individuals from an immunological perspective and attempt to make a case for prioritising COVID-19 vaccination for those living with HIV-1 in Sub-Saharan Africa (SSA) countries like Malawi as one way of minimising the impact of COVID-19 in these countries.

## Introduction

Coronaviruses have been in existence since time immemorial and six coronaviruses are known to cause disease in humans (1, 2). Four of these human coronaviruses (hCoV), 229E, HKU1, NL63 and OC43, cause infections attributed as the common cold and are endemic in different parts of the world (1). However, the other two have been major health concerns with severe acute respiratory syndrome coronavirus (SARS-CoV) and the Middle East respiratory syndrome coronavirus (MERS-CoV) emerging in 2002 and 2012, respectively (3).

The first case of COVID-19 (coronavirus disease) caused by SARS-CoV-2 was reported to the World Health Organisation (WHO) by the Chinese authorities on 31st December 2019 (4, 5). Since its emergence SARS-CoV-2 has proven to be more lethal than the other hCoV with the WHO declaring it a global pandemic in March 2020 (6, 7). Globally, as of October 2021, there have been over 240 million cases and over 4.8 million deaths (8, 9).

SARS-CoV-2 has however not spread evenly throughout the world with countries like Italy, the UK, the USA, Iran, Brazil and India being the most affected countries (9). A report published in November 2020 showed that SARS-CoV-2 incidences appeared low in most African countries recording less than 4% of the global cases and deaths but in a background of limited COVID-19 testing (10) with African countries conducting the least tests (8, 9). Furthermore, as of September 2021, SSA, which has a population of 1.15 billion (about 14% of the global population) (11), had only received 2% of the world’s COVID-19 vaccine supply with most of the countries not reaching the target of vaccinating 10% of their population (12). In this review we report on the impact COVID-19 on HIV-1-infected individuals from an immunological perspective. We emphasize the need to prioritise COVID-19 vaccination for HIV-1-infected individuals in order to reduce the burden of COVID-19 upon low-resource countries in SSA such as Malawi.

## COVID-19 in Malawi and Africa

The first COVID-19 cases in Malawi were confirmed in April 2020 (13) and these were three cases, one index case and two local transmission cases. As of 17^th^ October 2021, there had been 61,716 confirmed cases and 2,292 deaths (14, 15). Of the confirmed cases, the average age was 36 years and 66.9% were male (14). Among the confirmed COVID-19 deaths, the average age was 56.7 years and 82.5% were male (9, 14). While initial COVID-19 cases were primarily imported, the number of local transmissions surpassed imported cases by July 2020 (14). The lower than expected burden of COVID-19 on the African continent massively contradicted various projections which had been calculated at the onset of the pandemic (16, 17). Various theories, ranging from genetics to BCG vaccine administration, lower testing rate compared to other countries, have been proposed to explain the lower than expected burden of the disease on the continent (18–21) but these are yet to be proven.

Although the vast majority of those who get infected with COVID-19 remain asymptomatic or merely manifest mild flu-like symptoms, some individuals develop life-threatening severe disease and require hospitalization or long COVID-19 disease (15, 22, 23). For the original variant first detected in Wuhan, China (4, 5), the main risk factor for developing severe COVID-19 disease and COVID-19-related mortality was age with those aged 65 years or more being at higher risk (24). Other risk factors included being male, having other underlying conditions such as diabetes, severe asthma, smoking, blood group and obesity (24, 25). However, lately some variants, especially the Delta, are causing disease even amongst the young (26).

**Figure 1:**
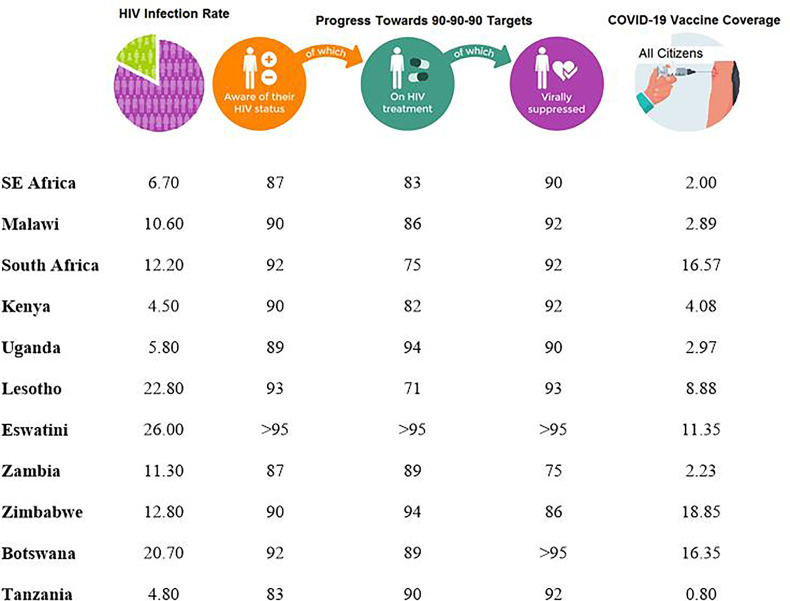
Progress made by various countries in the Sub-Saharan Africa towards achieving the 90-90-90 Targets as of 2020 (70) and COVID-19 percentage coverage for individuals who had been fully vaccinated, regardless of HIV status, in each country as of September 2021 (71).

Although Malawi as a country has lower rates of COVID-19 infection compared to other African countries, HIV-1/AIDS prevalence is still high. With a population of close to 17 million, the HIV-1 prevalence amongst individuals aged between 15 and 64 years is 10.6% (27–29). This rate is quite similar to other African countries like South Africa with the national prevalence rate of 12.2% with approximately 6.4 million living with HIV-1 (30) but lower than that of Eswatini (formerly Swaziland) which is estimated at 26% (31) (**Figure 1**).

Recent studies have not established any association between being HIV-1-infected and being more vulnerable to contracting COVID-19 (32), but have shown that being infected with HIV-1 is a risk factor for developing severe COVID-19 and for COVID-19-related mortality (33–35). It is not known how many of the cases of COVID-19 and COVID-related deaths in Malawi were people living with HIV-1 (15). Since the pandemic started over 90 vaccine candidates have been developed. Malawi, like most other African countries, started receiving the Vaxzevria/Oxford-AstraZeneca vaccine through the COVAX initiative with 360,000 doses delivered in March 2021 with more doses in August 2021 supplemented with doses of the Johnson and Johnson’s Jansen vaccine (36). Despite this, by 17th October 2021 only 3.01% Malawians had been fully vaccinated (12).

## Immunology of COVID-19, Virus Mutation, and Vaccines

SARS-CoV-2 gains entry into human cells by binding to the Angiotensin Converting Enzyme 2 (ACE2) receptor, which is expressed by various cells including lung epithelial cells (37). Entry of virus into the body triggers the host immune system starting with the innate immune cells which recognise the molecular patterns associated with the virus (38). Two recent reviews (39, 40) provide detailed outlines of the different immune components that are involved in the human response against SARS-CoV-2 infection.

At the innate immune response level, a significant increase of monocytes and macrophages has been observed in individuals infected with SARS-CoV-2 with the macrophages infiltrating the lungs and secreting inflammatory cytokines such as IL-6 and IL-1β (41). Infected individuals have also been observed to have a suppressed type 1 IFN response, which is fundamental in the fight against viral infections (41). In contrast, other immune cells such as eosinophils were observed to be much lower than normal in those infected with SARS-CoV-2 (42) whereas mast cells were reported to secrete inflammatory cytokines such as IL-1, IL-6, IL-33 and other mediators such as histamine and protease (43, 44). Decreased cell counts of natural killer (NK) cells were observed in individuals infected with SARS-CoV-2 and these predominantly expressed an exhaustion phenotype (45). Failure of the innate immune response to eliminate the virus will normally lead to the activation of the adaptive immune system with T and B cells involved. The CD4+ and CD8+ T cell responses target all parts of the SARS-CoV-2 proteome with CD4+ T cells dominating the response (46, 47).

The severity of COVID-19 patients has been associated with a skewed CD4+ T cell response to cytotoxic CD4+ T follicular helper (Tfh) cells, reduced regulatory T cells (48) and a less coordinated CD4+ and CD8+ T cell response (47). In infected individuals B cell subsets were observed to be lower than normal but the actual amount of SARS-CoV-2 specific IgG produced was high (49). Antibodies are thought to be protective with convalescent plasma and neutralizing monoclonal antibodies used as treatment for COVID-19 patients (50, 51). However, the antibody titre levels against the receptor binding domain and anti-spike neutralizing antibodies were reportedly higher in more severely affected patients compared to mildly ill patients (52). This may also be a consequence of prolonged infection.

>The efficacy levels reported for each vaccine type are those observed against the original strain of SARS-CoV-2 initially detected in Wuhan, China. However, since then several different variants of the virus have emerged (53). The Alpha variant, first documented in the UK in September 2020, became dominant, but has now been superseded by the Delta variant that emerged from India in October 2020 (53). Other variants of concern (VOC) are the Beta variant and the Gamma variant (53). The emergence of these new variants has questioned whether the various COVID-19 vaccines currently in use would maintain their efficacies.

One recent study reported reduced efficacy of the Pfizer vaccine against the Alpha and Beta variants (54) with others hypothesizing that some of the vaccines will drastically lose their efficacy against the new variants (55). The clinically approved human monoclonal antibody treatments, bamlanivimab and etesevimab, were unable to neutralize the Beta variant (56). Furthermore, both the Beta and Delta variants were more resistant to neutralization to sera from Moderna/Spikevax, Comirnaty/Pfizer-BioNTech and Vaxzevria/Oxford-Astra Zeneca vaccines than the Alpha variant (57).

One of the main concerns with the COVID-19 vaccines has been whether their efficacy could be affected when administered to HIV-1-infected individuals due to their immunocompromised status (58). However, results of two recent studies investigating the efficacy of the AstraZeneca COVID-19 vaccine in the UK and South Africa reported that there were no differences in terms of vaccine-related antibody or T cell-mediated responses between HIV-1-infected participants and HIV-1-negative controls at any stage of the vaccination process (58, 59). The studies also showed that antibody responses were not affected by the CD4 T cell count in the HIV-1-infected individuals (58, 59). Similar results were reported by a group working on a nanoparticle vaccine, Novavax, although the researchers still propose that more work needs to be done in HIV-1-infected individuals with CD4 T cell counts lower than 350 cells/μl blood and with detectable viral loads (60).

## HIV-1 and Immune Dysfunction

It has been shown that years of untreated HIV-1 infection before commencing combination antiretroviral treatment (cART) can substantially affect the length of time the immune system needs to fully recover (61). Thus, it is beneficial for HIV-1+ patients to start cART as early as possible (62). An environment of activation, dysfunction and inflammation pervades the immune system during untreated HIV-1 infection. Chronic immune activation of T cells occurs through persistent depletion and expansion of T cells accompanied with over-expression of CD38 and HLA-DR (62). In B cells there is an over-production of autoantibodies, increased expression of activation markers but also dysfunctional responses to T cell help and a loss of memory B cells (63). The innate immune system shows raised levels of IL-1, IL-6, TNF and C-reactive protein (CRP) amongst other things (62). Meanwhile CD4+ Tfh cells are expanded in untreated HIV-1, which leads to changes in certain B cell populations, an increase of germinal B cells, fewer memory B cells and more BCL6 transcriptional repressor (64).

Furthermore, B cell vaccine responses to influenza, HPV and yellow fever are attenuated in HIV-1 patients (65–67). During HIV-1 infection, there is also loss of the mucosal barrier leading to microbial translocation demonstrated through the increased levels of soluble CD14 and soluble CD163 (62). This drives activation of the innate immune system, such as macrophages along with abnormally raised levels of pro-inflammatory cytokines such as IFN-α, TNF-α, IL-1, IL-6 and IL-18 (62). One of the roles of plasmacytoid DCs (pDC) is to produce IFN-α and IFN-β during early stages of a viral infection. Unfortunately, HIV-1 infection impairs the function of pDC and reduces their frequency (68).

The introduction of cART in the late nineties has had a major impact on the lives of millions of HIV-1+ patients increasing life expectancy to non-infected levels. However, depending largely on the CD4+ T cell count before cART commencement (61), full immune restoration is not always attained such that as many as 16% may not attain CD4+ T cell counts greater than 200 cells/μl blood after four years of treatment (69).

According to UNAIDS, 83% (with a range of 60-92%) of HIV-1-infected individuals in eastern and southern Africa had access to cART in 2020 (70). For Malawi, it was estimated that 88% had access to cART and 92% had suppressed viral loads. The respective percentages for some selected SSA countries are presented in **Figure 1**. Based on these figures, it is clear that a significant population of HIV-1+ patients in SSA are potentially vulnerable to further HIV-1 associated opportunistic infections and other pathogens such as SARS-CoV-2.

While cART does have an effect on reducing T cell activation, it does not completely abolish all chronic immune activation as there are still elevated levels of IL-6, CRP, D-dimer and sCD14 (72). The frequency of Tfh cells drops to normal levels under cART but there is an over-representation of germinal centre B cells and an under-representation of memory B cells (72). Meanwhile, the innate immune functionality of pDC remains affected in patients on cART, which may compromise the anti-viral response (68). Myeloid dendritic cells frequencies appear normal before and during cART but their ability to skew towards to Th1 responses is impaired (73).

Hearps and colleagues showed that monocytes have impaired phagocytic activity in cART patients and they resemble those of elderly HIV-1-negative subjects (74).

Natural killer (NK) cells play a major role against viral pathogens producing IFN-γ and killing virally-infected cells through antibody-dependent cellular cytotoxicity (ADCC). However, in HIV-1+ patients their ability to performed ADCC is compromised and persists under cART (75). Therefore, all these factors may affect the ability of HIV-1+ patients to respond to further viral infections including SARS-CoV-2 especially if the CD4+ T cell counts of the affected individuals have not yet “normalized” in the course of being on cART.

Specifically, if plasma levels of IL-6 are already elevated in cART patients, some have speculated that such individuals would be more likely to get more severe symptoms of COVID-19 (76). One of the major risk factors of mortality in COVID-19 is age (24) and it has been suggested that many of the immuno-cellular disturbances associated with HIV-1 infection have similarities with immune systems in the elderly (77). The hallmarks of immune systems in the elderly consist of declining frequencies of naive T cells and hematopoietic progenitor cells with heightened levels of inflammation, which all have parallels with HIV-1 affected immune systems (77).

Although, multiple factors contribute to pathology associated with COVID-19, an aged immune system because of a HIV-1 infection may not be beneficial. Currently, several developed countries are already administering COVID-19 vaccine booster shots to individuals aged 65 years and above and those who are immunocompromised (78). Considering the similarities of the immune systems, comparable requirements might also be essential for HIV-1-infected patients.

## COVID-19 in HIV-1-Infected Individuals

As mentioned earlier, no study so far has established any link between being HIV-1-infected and being more vulnerable to contracting COVID-19 (32). What is known though is that being infected with HIV-1 is a risk factor for developing severe COVID-19 (79) and for COVID-19 related mortality (33–35). Furthermore, a recent case report in South Africa revealed the development of over ten new SARS-CoV-2 variants in one individual who presented with untreated HIV-1 infection and had been co-infected with SARS-CoV-2 (80). The authors proposed that untreated HIV-1 infection might provide a fertile environment that favours intra-host mutation of SARS-CoV-2 (80).

With a seemingly waning efficacy of the current vaccines against some of the SARS-CoV-2 variants (54–57), a scenario whereby the current vaccines become ineffectual with time is a real possibility. The recent reports of individuals who had been infected with two different SARS-CoV-2 variants (81–83) suggest that it is possible for some individuals to be concurrently infected by more than one variant of SARS-CoV-2 and subsequently more virulent variants could emerge through recombination (84). With COVID-19 vaccines proving to be just as effective in HIV-1-infected individuals, prioritising inoculation of this group might be one of the ways of mitigating variant development. The WHO provides guidelines on the prioritization of vaccine administration if supplies are limited (85). The potential risk of intra-host mutation development in HIV-1-infected individuals reported in South Africa (80) may serve as an additional justification for the WHO to move this population group further up the COVID-19 vaccination priority list. If this observation is repeated in other studies involving both untreated and treated HIV-1-infected individuals in countries where HIV/AIDS prevalence is high, countries might wish to prioritise all HIV-1-infected individuals to be vaccinated against SARS-CoV-2 in order to keep the variants development in check.

## Challenges Ahead

As long as there is significant community transmission taking place, the virus will continue to mutate and variants emerge (84). One such variant, C.1.2, was first detected in May 2021 in Mpumalanga South Africa and by August 2021 it had spread to various provinces in the country (84). Although much about the C.1.2 variant in terms of virulence and transmissibility is yet to be fully elucidated, its discovery emphasises the point that in countries where a good proportion of the population is unvaccinated, VOCs will continue to arise posing a threat to the world at large. As it has been shown with the Delta variant, the efficacy of the current COVID-19 vaccines against the new variants tends to be compromised (54, 55). In the event that a new variant emerges on the global scene, which is completely resistant to all current vaccines, it would derail the fight against SARS-CoV-2 and downgrade the gains so far attained. As more countries gradually but cautiously lift travel bans from high-risk countries, the risk of new vaccine-resistant variants spreading to different parts of the world remains high (86).

Of major concern is the recently observed disparity in vaccine coverage between developed countries, which have already attained over 75% vaccine coverage, and most African countries (**Figure 1**) which, on average, have only achieved 2% coverage (87). This so-called “vaccine apartheid” phenomenon could be exacerbated if the current trend of more developed countries proceeding with the implementation of proposed “booster” or third jab (88, 89). These vaccines could have been more beneficial, and more effective against the pandemic, if administered either as first or second jabs in developing countries. The recent report by WHO (90) on some developed countries having even a greater access towards the vaccines originally meant for developing countries under the COVAX initiative makes this scenario even worse. Meanwhile the ensuing low COVID-19 vaccination coverage in Africa (71) still provides a potentially conducive environment for the development of new SARS-CoV-2 variants.

While it is clear that the majority of unvaccinated individuals survive primary infection without the need for hospitalization (91) what remains to be fully elucidated are the specific immune correlates of protection against SARS-CoV-2 (92–94). The pandemic will continue until further studies reveal the veritable correlates of protection against SARS-CoV-2 and/or the whole world is immunized, including those most vulnerable to severe COVID-19 such as those living with HIV-1. Waning vaccine-induced antibodies (95) and emerging new variants (54, 55, 79) will inadvertently prolong the period during which vaccine booster jabs and vaccines against VOC will be required.

The introduction of the Extended Program on Immunization (EPI) by WHO back in 1974 has been one of the success stories in the region (96). Given, the necessary support, SSA countries are capable of contributing substantially to the global fight against COVID-19.

## Author Contributions

Both authors contributed equally to this work in writing, reviewing, and editing.

## Funding

WM is funded by the Malawi University of Science and Technology and ML is currently funded by the Wellcome Trust [Grant Number: P86433].

## Conflict of Interest

The authors declare that the research was conducted in the absence of any commercial or financial relationships that could be construed as a potential conflict of interest.

## Publisher’s Note

All claims expressed in this article are solely those of the authors and do not necessarily represent those of their affiliated organizations, or those of the publisher, the editors and the reviewers. Any product that may be evaluated in this article, or claim that may be made by its manufacturer, is not guaranteed or endorsed by the publisher.
